# Manganese(ii) promotes prebiotically plausible non-enzymatic RNA ligation reactions†

**DOI:** 10.1039/d4cc01086h

**Published:** 2024-05-30

**Authors:** Ziwei Liu, Clancy Zhijian Jiang, Andrew D. Bond, Nicholas J. Tosca, John D. Sutherland

**Affiliations:** a MRC–Laboratory of Molecular Biology, Francis Crick Avenue, Cambridge Biomedical Campus Cambridge CB2 0QH UK; b Department of Earth Sciences, University of Cambridge Downing Street CB2 3EQ UK zwl25@cam.ac.uk; c Yusuf Hamied Department of Chemistry, University of Cambridge Lensfield Road CB2 1EW UK

## Abstract

Using different prebiotically plausible activating reagents, the RNA ligation yield was significantly increased in the presence of Mn(ii). The mechanism of the activation reaction has been investigated using 5′-AMP as an analogue.

## Abstract 2

Using different prebiotically plausible activating reagents, the RNA ligation yield was significantly increased in the presence of Mn(II).

## Graphical abstract

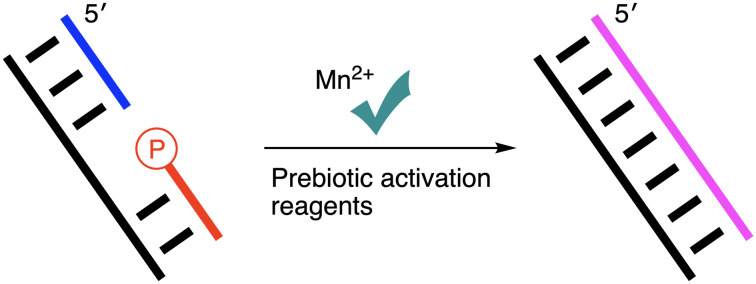

On early Earth, before the advent of DNA and proteins, RNA is widely thought to have stored genetic information and catalyzed chemical reactions; this has been described as the RNA world hypothesis. However, RNA must have reached a specific length in order to have served either function. RNA ligation from small pieces of RNA fragments was likely the means of assembling longer functionalized RNA strands. Magnesium and other metal ions have been used to assist RNA ligation by deprotonating the 3′-hydroxyl nucleophile,^1^ but the potential role of Mn(ii) in this context has received little attention despite geochemical and biochemical considerations suggesting that it may have been available in prebiotic aqueous environments.

Manganese is the 10th most abundant metal in the Earth's crust where it occurs almost exclusively as Mn(ii) through substitution for Fe(ii) in igneous and metamorphic minerals.^2^ Although it is typically present in crustal rocks at approximately 0.1 wt% as MnO, manganese concentrations can increase several-fold through fluid-magma interactions during the late stages of magma crystallisation.^3^ Mn(ii) would have been readily leached from igneous and metamorphic rocks under weakly acidic conditions, and Mn(ii) is soluble under the anoxic conditions thought to characterise the prebiotic Earth;^4,5^ high potential oxidants are required to facilitate its transformation to Mn(iii) or Mn(iv).^3,4,6^ This suggestion is consistent with the first appearance of substantial sedimentary Mn-deposits just after the initial appearance of O_2_ in Earth's atmosphere,^7^ and with a wealth of evidence from Archean-aged (>2.5 billion years old) rocks formed in shallow and deep marine environments.^8–11^ These latter data collectively indicate that Mn(ii) oxidation was negligible on early Earth, with removal from the oceans facilitated only by trace incorporation into CaCO_3_. These considerations also apply to non-marine prebiotic environments such as lakes and other standing water bodies, where Mn(ii) concentrations may have been even higher. First, evaporation of terrestrial waters would have led to significant increases in Mn(ii) concentrations, with maximum Mn(ii) concentrations principally controlled by partitioning into carbonate minerals, the formation of which is dependent on alkalinity, atmospheric P_CO_2__, and cation concentration.^4^ Specifically, in the presence of Ca, Ca–Mn carbonates may have limited Mn(ii) concentrations,^12,13^ but in Ca-poor systems, which are thought to have been common in alkaline lake settings,^14^ Mn(ii) removal into Mn-carbonate minerals would have served as the most likely control on maximum concentrations.^13^ The common supersaturation of some anoxic waters with respect to Mn-carbonates on the modern Earth,^15,16^ and the slow kinetics of Mn-carbonate precipitation,^16^ together suggest that high degrees of supersaturation, and therefore high Mn(ii) concentrations (*i.e.*, at the mM level) may have been common in alkaline lakes on the prebiotic Earth.

In addition, from its role in catalyzing water oxidation within photosystem II, to its involvement in the synthesis and activation of several enzymes, manganese is an essential element in modern biology. Although the details of how Mn(ii) became involved in modern biochemistry are poorly understood, a number of observations have shown that Mn(ii) can mediate the catalytic activity of some RNA ligases. For example, the crystal structure of *Pyrococcus horikoshii* RtcB,^17^ an RNA ligase joining either 2′,3′-cyclic phosphate or 3′-phosphate termini to 5′-hydroxyl termini, indicates that RtcB catalysis is dependent on GTP and Mn(ii). Additionally, the RNA ligase within *Deinococcus radiodurans* (DraRnl), which can seal a 3′-OH/5′-phosphate nick in a duplex RNA, requires Mg(ii) or Mn(ii) as a cofactor, but requires lower concentrations of Mn(ii) relative to Mg(ii) to achieve equivalent activity.^18^ A similar RNA ligase has been found in *Naegleria gruberi* (NgrRnl),^19^ which features a two-metal mechanism of lysine adenylation, where Mn(ii) can occupy both metal centres.^20^ Accordingly, we wondered if Mn(ii) could promote RNA ligation using prebiotically plausible activating reagents.

*N*-Cyanoimidazole (NCI, Scheme 1a) has been used for chemical RNA ligation,^21–24^ but the mechanism of the role of manganese in this reaction has not been investigated. Here we propose the intermediates for this reaction in Scheme 1b. Manganese is an azophilic metal which can be chelated by the imidoyl phosphate moiety through oxygen and nitrogen *via* a 6-membered ring. This effect increased the reactivity of the imidoyl phosphate moiety to nucleophilic attack by either imidazole or hydroxyl group leading to 5′-phosphoramidate or ligation product, respectively. Phosphorimidazolide-RNAs are subsequent intermediates, the nitrogen of the phosphorimidazolide moiety potentially binding to Mn(ii), which can increase the reactivity. After 24 hours of incubation at room temperature, the resulting solution was diluted to a loading buffer and then analysed by polyacrylamide gel electrophoresis (PAGE) (ESI,† Fig. S1). Compared with Mg(ii), the yield of the ligation product is 9-fold higher using Mn(ii). This indicates that Mn(ii) can increase the efficiency of phosphorimidazolide pre-activated RNA templated nicked-duplex ligation. This is because the nitrogen of phosphorimidazolide can be complexed with manganese, which increases the reactivity of phosphorimidazolide (Scheme 1b).

**Scheme 1:**
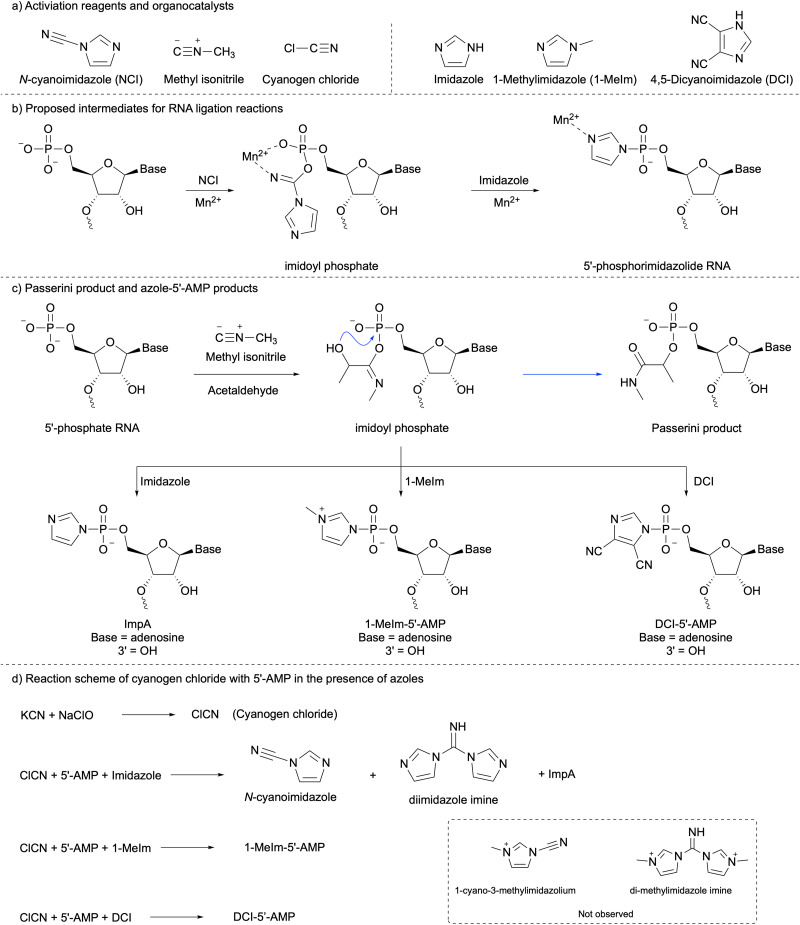
(a) Chemical structures of activation reagents and organocatalysts. (b) Proposed intermediates for RNA ligation reactions. (c) Passerini product and azole-5′-AMP products. (d) Reaction scheme of cyanogen chloride with 5′-AMP in the presence of azoles.

Methyl isonitrile (Scheme 1a), a prebiotically plausible activating reagent, has recently been used for mononucleotide activation, RNA monomer extension, and RNA ligation reactions with/without aldehyde.^25–29^ Here we tested the RNA ligation reaction using some organocatalysis with methyl isonitrile as an activating reagent. Among these organocatalysis, the combination of 4,5-dicyanoimidazole (DCI) and 1-methylimidazole (1-MeIm) stood out from others (ESI,† Fig. S4, line d). The reaction between methyl isonitrile and phosphate in the presence of aldehyde also generates products through the Passerini reaction (Scheme 1c, blue arrow),^30,31^ but imidazole can interrupt this reaction intermolecularly.^25^ Our results show that significant suppression can be attained in the presence of DCI (Experimental part and discussion in ESI†).

Cyanogen chloride (Scheme 1a) has been used as a nucleotide activation reagent,^32^ but not as an RNA ligation reagent. Thus, we tested whether cyanogen chloride, generated from sodium hypochlorite and potassium cyanide, could serve as an effective ligation reagent *in situ*. First, we tested which organocatalysts could help the ligation reaction in the presence of Mg(ii). After 2 hours of incubation with 1-MeIm or a mixture of 1-MeIm and DCI, ligation products were formed in comparable yield (ESI,† Fig. S9, lines b and d), which was much higher using purine instead of 1-MeIm (ESI,† Fig. S9, line c). Similar results have been observed after 24 hours. This indicates that 1-MeIm was the best catalyst for cyanogen chloride ligation. Second, we compared the efficiency of Mg(ii) with Mn(ii) for the nicked duplex RNA ligation reaction with cyanogen chloride. After 2 hours of incubation (Fig. 1 line b and c), the yield of ligation with Mg(ii) was 17%, but with Mn(ii), the ligation yield increased markedly to 63%. After 24 hours of incubation (Fig. 1 lines d and e), both yields were not improved significantly, which was because the activating reagent was consumed in the first few hours. We then tested if imidazole could be used instead of 1-methylimidazole for the RNA ligation reaction. The same reactions were repeated using different ratios between imidazole and 1-methylimidazole. After 2 hours of incubation with imidazole (ESI,† Fig. S10 line b–e), all yields of ligation products were suppressed; higher imidazole concentrations in the reaction mixture resulted in slower reaction kinetics. After 24 hours (ESI,† Fig. S10 line f–i), the final yields of all reactions were similar, with 20% imidazole in the solution, and a ligation yield of nearly 90%. We repeated the same reaction with Mg(ii) instead of Mn(ii) (ESI,† Fig. S12), and the ligation yield in all cases was significantly decreased.

**Fig. 1:**
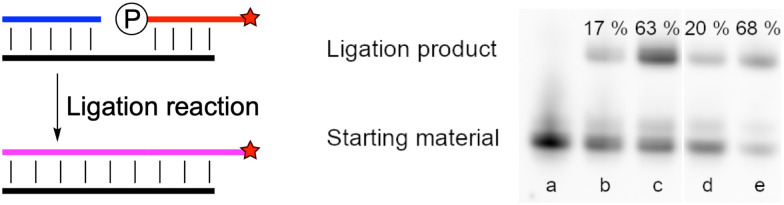
PAGE analysis of *in situ* nicked-duplex RNA ligation using 5′-pUACUGGCA-Cy3 (10 μM), 5′-GAGAACC (20 μM), 5′-CCAGUAGGUUCUC (10 μM), 1-MeIm (100 mM), KCN (50 mM), sodium hypochlorite (20 mM), pH value was 6, (a) before sodium hypochlorite was added; (b) with MgCl_2_ (10 mM), incubated for 2 hours; (c) with MnCl_2_ (10 mM), incubated for 2 hours; (d) same as b, incubated for 24 hours; (e) same as c, incubated for 24 hours.

To understand the difference in reactivity between imidazole or 1-MeIm with cyanogen chloride, 5′-AMP has been used as an analogue. The reaction products were analyzed by ^1^H- and ^31^P-NMR spectroscopy after a designated time interval. In the presence of imidazole, imidazolide-5′-AMP (ImpA) was not observed in ^31^P-NMR spectra (ESI,† Fig. S13) because the concentration of cyanogen chloride was not high enough to generate a detectable amount of ImpA under these conditions. In ^1^H-NMR spectra (ESI,† Fig. S14), diimidazole imine (∼2.6 mM, (Scheme 1d)) and NCI (∼1 mM) were observed within 5 minutes. After 12 hours, a trace amount of diimidazole imine remained. To demonstrate the formation of diimidazole imine and ImpA, the reaction of 5′-AMP (10 mM), and NCI (100 mM) in an imidazolium nitrate buffer (200 mM, pH = 6.2) was investigated. Diimidazole imine was formed in the first few minutes (ESI,† Fig. S15). After 1 day of incubation, a small amount of diimidazole imine remained. In this reaction, the amount of NCI was 10-fold of 5′-AMP, thus, in ^31^P-NMR spectra (ESI,† Fig. S16), ImpA was observed. The maximum yield of ImpA was about 11% after 12 hours of incubation. As the pH of the reaction was increased, ImpA was more stable at higher pH. This explains why ImpA was not observed at the beginning, but it appeared later even with lower concentrations of NCI and diimidazole imine. The result of the reaction with 1-MeIm was completely different. 1-Cyano-3-methylimidazolium or di-methylimidazole imine (Scheme 1d) was not observed by ^1^H-NMR spectroscopy (ESI,† Fig. S17), instead, 1-MeIm-5′-AMP was observed, which was confirmed by ^31^P-NMR (Fig. 2). These results indicated the cyanogen chloride ligation with imidazole was slow because of the slow formation of imidazolide-RNA formation. The slow formation of imidazolide-RNA can still be improved by Mn(ii) for the RNA ligation reaction (ESI,† Fig. S1), thus, after 24 hours of incubation, the yield was 69% (ESI,† Fig. S10, line i). The cyanogen chloride ligation with 1-MeIm was fast as the 1-MeIm-RNA was instantly formed, which was a good candidate for RNA ligation.^33^

**Fig. 2:**
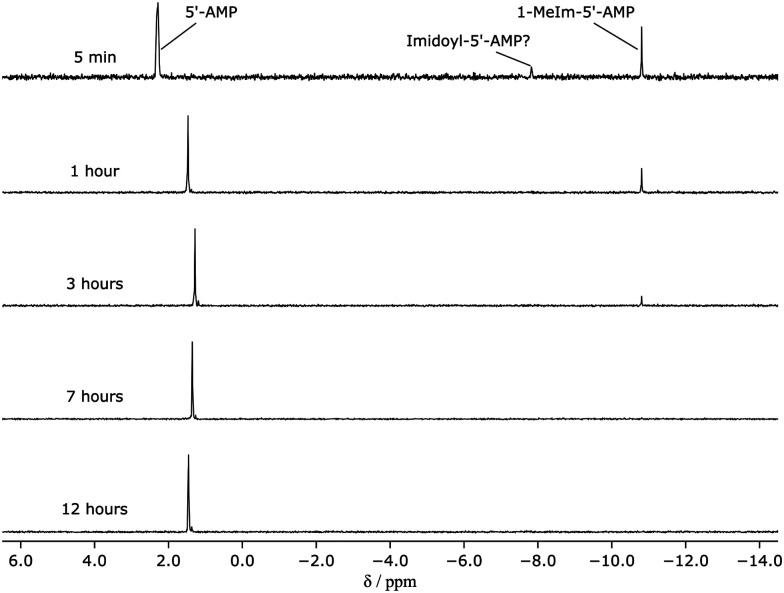
Stacked ^31^P-NMR spectra of a solution of 5′-AMP (10 mM), 1-MeIm (100 mM), potassium cyanide (50 mM), sodium hypochlorite (20 mM). The chemical shift drift of the signal for 5′-AMP was caused by changing pH.

Lastly, we compared the ligation efficiency between cyanogen chloride and *N*-cyanoimidazole with Mn(ii). The yield of nicked-duplex templated RNA ligation using only cyanogen chloride was 72% after 24 hours incubation (ESI,† Fig. S11 line b), the yield of RNA ligation using only NCI was 78% (ESI,† Fig. S11, line e). The cyanogen chloride ligation followed by NCI ligation gave an 87% yield. These results indicated the combination of cyanogen chloride, Mn(ii) and imidazole or 1-MeIm was an excellent activating reagent for RNA ligation.

We have demonstrated that Mn(ii) is more effective than Mg(ii) in enhancing templated RNA nicked-duplex ligations when utilizing pre-activated phosphorimidazolide-RNA. Subsequently, we conducted experiments involving templated RNA nicked-duplex ligation using three different activating reagents, employing various organocatalysis methods with either Mg(ii) or Mn(ii). In the presence of only methyl isonitrile as the activating reagent, 1-MeIm, DCI, and Mn(ii) were all essential to achieve the highest ligation yield. When using methyl isonitrile and acetaldehyde, both 1-MeIm and DCI were effective in significantly suppressing the Passerini reaction on mononucleotides. However, only DCI was capable of suppressing the Passerini reaction in RNA. Furthermore, the addition of Mn(ii) substantially increased the ligation yield when using cyanogen chloride, which was generated *in situ* through the reaction of hypochlorite and cyanide. From these results we may imagine that Mn(ii) could have played an important role in the emergence of RNA molecules of sufficient length to serve as both catalysts and repositories of genetic information, but only if Mn(ii) was available in sufficient quantities in prebiotic environments. This research was supported by the Medical Research Council (MC_UP_A024_1009 to J. D. S), the Simons Foundation (290362 to J. D. S) and the Leverhulme Centre for Life in the Universe, the Leverhulme Trust (RC-2021-032 to N. J. T). The authors thank Benjamin Tutolo, Jack Szostak, Dimitar Sasselov and all J. D. S. group members for fruitful discussions.

## Conflicts of interest

There are no conflicts to declare.

## Supplementary Material

CC-060-D4CC01086H-s001

CC-060-D4CC01086H-s002
