# Supplementary material for: Manganese(ii) promotes prebiotically plausible non-enzymatic RNA ligation reactions
Source: Chem Commun (Camb). 2024 May 30;60(51):6528–31. doi: 10.1039/d4cc01086h (PMC11189027; doi:10.1039/d4cc01086h)
Supplement: CC-060-D4CC01086H-s001 [file CC-060-D4CC01086H-s001.pdf]

Electronic Supplementary Material (ESI) for Chemical Communications.
This journal is © The Royal Society of Chemistry 2024

Supplementary Information

Manganese (II) promotes prebiotically plausible non-enzymatic RNA ligation reactions

Ziwei Liu[1,2,*], Clancy Zhijian Jiang[2], Andrew D. Bond[3], Nicholas J. Tosca[2], and John D. Sutherland[1]

[1]MRC – Laboratory of Molecular Biology, Francis Crick Avenue, Cambridge Biomedical Campus, Cambridge CB2 0QH, United Kingdom.
[2]Department of Earth Sciences, University of Cambridge, Downing Street, CB2 3EQ, United Kingdom.
[3]Yusuf Hamied Department of Chemistry, University of Cambridge, Lensfield Road, CB2 1EW, United Kingdom.
[*]Corresponding Author: zwl25@cam.ac.uk

Table of contents

Prebiotic synthesis pathway of organocatalysts 3

Passerini reaction in RNA reaction suppressed by DCI 3

General methods 4

Chemical synthesis of RNA oligomers 4

General procedure for desalting of RNA by ethanol precipitation 5

Chemical synthesis of 5′-phosphorimidazolide RNA oligonucleotide 5

General procedure for oligonucleotide ligation reactions using methyl isonitrile 5

General procedure for oligonucleotide ligation reactions using methyl isonitrile and acetaldehyde 6

General procedure for oligonucleotide ligation reactions using sodium hypochlorite and potassium cyanide 6

Comparison ligation reactions between cyanogen chloride and NCI 6

X-ray crystallography for 1-methyladenine : 4,5-dicyanoimidazole: H_2O 7

Supplementary figures S1-S16 and table S1-S2 8

Prebiotic synthesis pathway of organocatalysts

A prebiotically plausible synthesis of DCI involves heating cyanide in formamide[1], these conditions also generate adenine. 5-aminoimidazole-4-carbonitrile (AICN) is an intermediate in the synthesis of adenine under these conditions and has thus also been investigated in the context of ligation chemistry. In the presence of DCI, methyl isonitrile reacted with AICN, generating 1-methyladenine (Fig. S2), which co-crystallized with DCI (Fig. S3, Table S1). Hypoxanthine has been reported as a product resulting from adenine diazotization followed by hydrolysis[2,3].

Passerini reaction in RNA reaction suppressed by DCI

We tested the formation of different azolide-5′-AMPs using methyl isonitrile and acetaldehyde as activating reagents. Because Mn (II) is a paramagnetic metal ion which can interfere with NMR analysis, Mg (II) was used instead for these experiments and the results were analyzed by ^{31}P-NMR spectroscopy. 5′-AMP (10 mM), $MgCl_2$ (10 mM), DCI and/or 1-MeIm were dissolved in water and adjusted to pH 6, and acetaldehyde (100 mM) and methyl isonitrile (100 mM) were added sequentially afterwards. The mixture was transferred to a sealed NMR tube and analyzed by ^{31}P-NMR spectroscopy at a pre-determined time. As shown in Fig. S5, in the presence of DCI, the yield of DCI-5′-AMP (Scheme 1c) was about 50 %, and it hydrolysed quite rapidly; after 1 hour, the yield of DCI-5′-AMP dropped to 8 %. The stability of 1-MeIm-5′-AMP (Scheme 1c) was different from DCI-5′-AMP. As shown in Fig. S6, the formation of 1-MeIm-5′-AMP was not complete after 5 minutes, and the maximum yield was 95 % after 1 hour of incubation. After 3 hours, there was 76 % of 1-MeIm-5′-AMP left, which indicated 1-MeIm-5′-AMP was much more stable than DCI-5′-AMP. Furthermore, we tested the formation and stability of azolide-5′-AMP in a mixed solution of DCI and 1-MeIm. As shown in Fig. S7, the yield of 1-MeIm-5′-AMP was 82 % and the yield of DCI-5′-AMP was 11 %; the reaction was finished in 5 minutes. Both 1-MeIm-5′-AMP and DCI-5′-AMP were hydrolysed afterwards, and after 3 hours almost all DCI-5′-AMP was hydrolysed, but 74 % of 1-MeIm-5′-AMP remained. The stability of 1-MeIm-5′-AMP was similar to the experiments performed without DCI. These results indicated the formation and degradation of 1-MeIm-5′-AMP were not affected by the addition of DCI. Interestingly, the Passerini reaction was completely suppressed by adding 1-MeIm and/or DCI. Encouraged by these results, we tested RNA ligation using methyl isonitrile and acetaldehyde as activating reagents and with different concentrations of DCI and/or 1-MeIm. As shown in Fig. S8, with a lower concentration of methyl isonitrile and acetaldehyde (Fig. S8, line a), both the ligation yield and the Passerini reaction yield were lower than with higher concentrations of activating reagents (Fig. S8, line b), but the ratio of ligation and Passerini reaction products was similar. With addi-

tional DCI (10 mM, Fig. S8, line c), both ligation and the Passerini reaction were not changed, but with an even higher concentration of DCI (100 mM, Fig. S8, line d), the ligation yield was increased and the Passerini reaction was suppressed. Similar reactions were repeated with $MnCl_2$ instead of $MgCl_2$, and the results were similar (Fig. S8, line e and f). Because the junction of the nicked-duplex of RNA is more crowded than a mononucleotide, the Passerini reaction in RNA reaction cannot be completely suppressed. Nevertheless, our results show that significant suppression can be attained in the presence of DCI. Furthermore, we have tested the effect of the concentration of Mn(II) (Fig. S8, line g and h). Even with 0.1 mM of Mn(II), the yield of ligation doesn't drop significantly.

General methods

Reagents and solvents were obtained from *Acros Organics, Alfa Aesar, Santa Cruz Biotechnology, Sigma-Aldrich, SYNTHON Chemicals GmbH & Co., KG* and *VWR International*. Reagents and solvents were used without further purification unless otherwise stated. Phosphoramidites for RNA synthesis were purchased from *Sigma-Aldrich* or *Link Technologies*. The Cyanine3 (Cy3) labelled oligonucleotides were purchased in HPLC-purified Na^+ form from *Integrated DNA Technologies*. The non-labelled oligonucleotide was synthesized using an ÄKTA oligopilot plus 10 instrument (*GE Healthcare*). A *Mettler Toledo* SevenEasy pH Meter S20 combined with a *Thermo Fisher* Scientific Orion 8103BN Ross semi-micro pH electrode was used to measure and adjust the pH to the desired value. NMR spectra (^{1}H, and ^{31}P) were acquired using a *Bruker* Ultrashield 400 Plus instrument or a *Bruker* Ascend 400 instrument operating at 400.13, and 161.97, respectively. Samples consisting of H_2O/D_2O mixtures were analysed using HOD suppression to collect ^{1}H-NMR spectroscopy data. Chemical shifts (δ) are shown in ppm. Mass spectra were acquired on an *Agilent* 1200 LC-MS system equipped with an electrospray ionization (ESI) source and a 6130 quadrupole spectrometer (LC solvents: A, 0.2 % formic acid in H_2O and B, 0.2 % formic acid in acetonitrile). Gel electrophoresis experiments using 20 % polyacrylamide and 8 M urea gels (8 cm $\times$ 8 cm $\times$ 1 mm) were typically run at 6 W in Tris/Borate/EDTA buffer. Fluorescence imaging was performed using an Amersham Typhoon imager (*GE Healthcare*) and quantified using Image Quant TL software (version 7.0). Oligonucleotide concentrations were determined by ultraviolet absorbance at 260 nm using a NanoDrop ND-1000 spectrophotometer.

Chemical synthesis of RNA oligomers

After automated synthesis, RNAs were first cleaved from the solid support by treating with 3 mL of a 1:1 mixture of NH_3 aqueous solution (28 % wt.) and CH_3NH_2 ethanol so-

lution (33 % wt.) at 55 ℃ for 90 min in a tube with a sealed cap. The solid was removed by filtration and washed with 50 % EtOH/H_2O. The solutions were combined and evaporated to dryness under reduced pressure. Silyl protecting groups were removed by treating the residues with 2 mL of a 1:1 mixture of triethylamine trihydrofluoride and DMSO at 65 ℃ for 3 hours in a tube with a sealed cap. After brief cooling at –32 ℃, 40 mL of cold 50 mM $NaClO_4$ in acetone was added to the solution to precipitate the oligoribonucleotides. The resulting mixture was centrifuged and oligoribonucleotides was dried by lyophilization. The RNA was redissolved in 5 mL of water and passed through a *Waters* Sep–Pak C18 Cartridge with 10 g sorbent. The cartridge was prewashed with 50 mL of acetonitrile then 50 mL of water before sample loading, washed with 150 mL of H_2O and 50 mL of 10 % aqueous acetonitrile. Eluates were checked for RNA content using a NanoDrop spectrophotometer. After lyophilizing, the resulting white powder was stored at –32 ℃ for future use.

General procedure for desalting of RNA by ethanol precipitation

Oligonucleotides were desalted by addition of 2 M imidazole nitrate solution (pH 6.2, 1/10 the volume of the aliquot taken from the reaction), followed by a 3 M sodium acetate solution (pH 5.2, 1/10 the volume of the aliquot taken from the reaction) and absolute ethanol (to a final concentration of 75 % (v/v)). The resulting mixture was kept at –20 ℃ for 3 h and then centrifuged for 30 min at 16,000*g*. The supernatant was removed, and the pellets were washed with 75 % (v/v) aqueous ethanol before additional centrifugation (10 min at 16,000*g*). The resulting pellets were air dried before being re-dissolved in water.

Chemical synthesis of 5′-phosphorimidazolide RNA oligonucleotide

A 5′-phosphorimidazolide RNA oligonucleotide was prepared by mixing EDC (1-Ethyl-3-(3-dimethylaminopropyl)carbodiimide, 80 mM), RNA (5′-pUACUGGCA, 1 mM), imidazole (80 mM) in aqueous solution (300 µL) at pH 6. After 2 hours of incubation at room temperature, RNAs were precipitated by pouring into a cold acetone solution (5 mL) of $NaClO_4$ (50 mM)[4].

General procedure for oligonucleotide ligation reactions using methyl isonitrile

To an aqueous solution of RNAs, organocatalysts, and divalent metal ion was added nuclease-free water to 8 µL, then methyl isonitrile (400 mM, 2 µL of a 2 M aqueous

stock solution) was added and the reaction was kept at room temperature. Aliquots of 3.0 µL were taken at the indicated time points and desalted by ethanol precipitation. For each aliquot, the resulting pellet was re-dissolved in 3.0 µL of nuclease-free water and 1.0 µL of the resulting solution was mixed with 4.0 µL of loading dye (95 % (v/v) 10 M urea in H_2O, 5 % (v/v) EDTA solution (0.5 M in H_2O, pH = 8.0), Blue bromophenol). The resulting mixture was analysed using polyacrylamide gel electrophoresis.

General procedure for oligonucleotide ligation reactions using methyl isonitrile and acetaldehyde

To an aqueous solution of RNAs, organocatalysts, and divalent metal ion was added nuclease-free water to 6 µL, then acetaldehyde (400 mM, 2 µL of a 2 M aqueous stock solution) was added, then methyl isonitrile (400 mM, 2 µL of a 2 M aqueous stock solution) was added and the reaction was kept at room temperature. Aliquots of 3.0 µL were taken at the indicated time points and desalted by ethanol precipitation. For each aliquot, the resulting pellet was re-dissolved in 3.0 µL of nuclease-free water and 1.0 µL of the resulting solution was mixed with 4.0 µL of loading dye (95 % (v/v) 10 M urea in H_2O, 5 % (v/v) EDTA solution (0.5 M in H_2O, pH = 8.0), Blue bromophenol). The resulting mixture was analysed using polyacrylamide gel electrophoresis.

General procedure for oligonucleotide ligation reactions using sodium hypochlorite and potassium cyanide

To an aqueous solution of RNAs, organocatalysis, potassium cyanide and divalent metal ion was added nuclease-free water to 6 µL, then sodium hypochlorite (20 mM, 4 µL of a 0.05 M aqueous stock solution) was added and the reaction was kept at room temperature. Aliquots of 1.0 µL were taken at the indicated time points was mixed with 4.0 µL of loading dye (95 % (v/v) 10 M urea in H_2O, 5 % (v/v) EDTA solution (0.5 M in H_2O, pH = 8.0), Blue bromophenol). The resulting mixture was analysed using polyacrylamide gel electrophoresis.

Comparison ligation reactions between cyanogen chloride and NCI

Three RNA components were mixed with imidazole (100 mM), potassium cyanide (50 mM), and $MnCl_2$ (10 mM) at pH 6. The resulting solution was separated into two parts, and to one of them sodium hypochlorite (20 mM) was added. Both mixtures were incubated at room temperature for 24 hours, 1 µL aliquot was diluted into a loading buffer, and to both of the remaining reaction solutions was added *N*-cyanoimidazole

(100 mM) before incubation at room temperature for another 24 hours. A 1 µL aliquot of the resultant solution was diluted into a loading buffer and then analysed by PAGE.

X-ray crystallography for 1-methyladenine : 4,5-dicyanoimidazole: H_2O

Single-crystal X-ray diffraction data were collected on a Bruker D8-QUEST diffractometer, equipped with an Incoatec IµS Cu microsource (λ= 1.5418 Å) and a PHOTON-III detector operating in shutterless mode. The crystal temperature was held at 180(2) K using an Oxford Cryosystems open-flow N_2 Cryostream. The control and processing software was Bruker APEX3. The structure was solved using SHELXT[5] and refined using SHELXL[5]. H atoms attached to C atoms were placed in idealised positions and refined as riding. H atoms attached to N or O atoms were located in the difference Fourier map and refined freely with isotropic displacement parameters.

Supplementary figures S1-S16 and table S1-S2

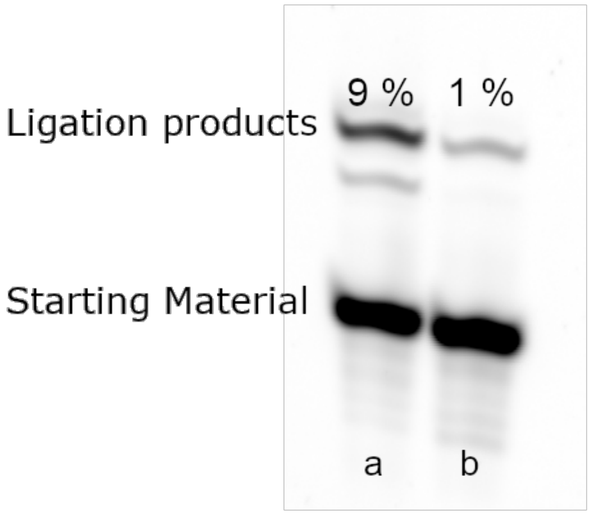

Figure S1. PAGE analysis of nicked-duplex RNA ligation using ImpUACUGGCA (20 µL), 5′-Cy3-CAGAGAACC (10 µL), 5′-CCAGUAGGUUCUC (10 µL) in imidazolium nitrate buffer (200 mM, pH = 6).
a) with $MnCl_2$ (10 mM);
b) with $MgCl_2$ (10 mM).

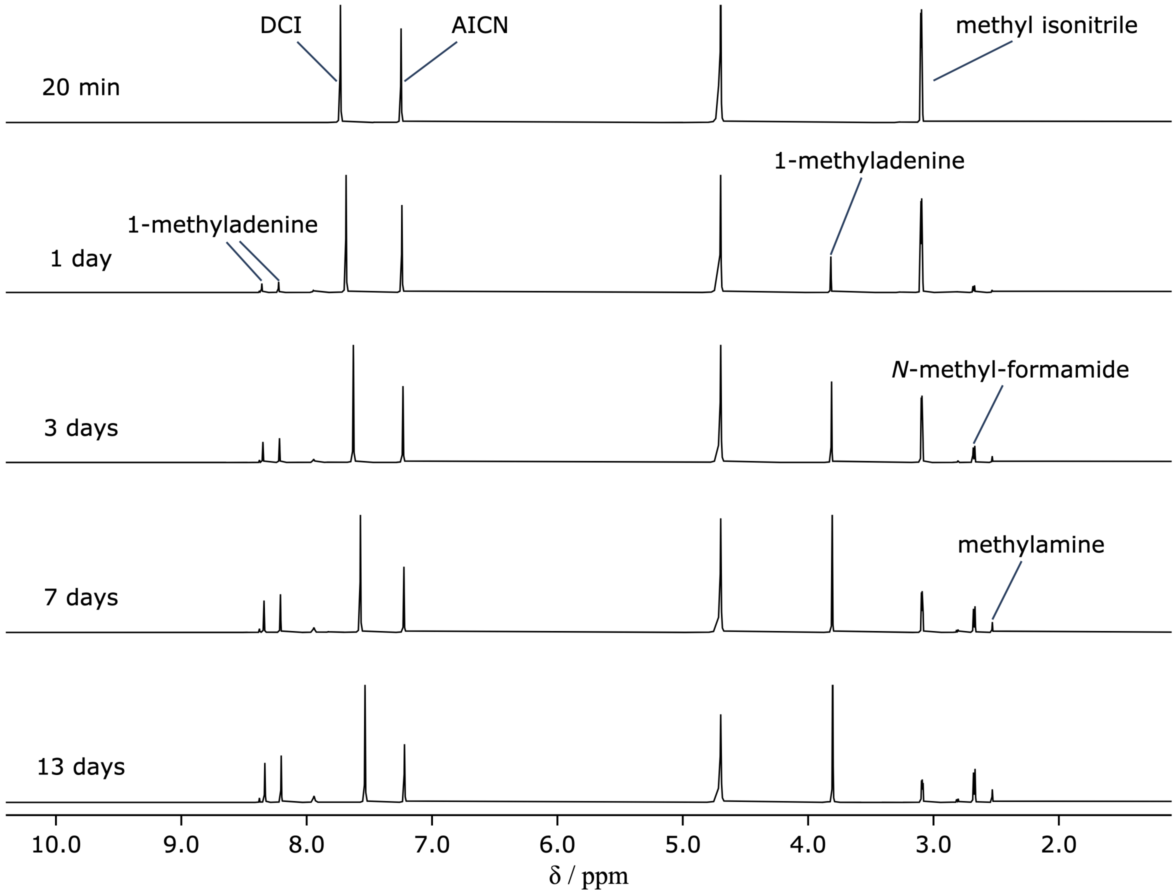

Figure S2. Stacked ^{1}H-NMR spectra of a solution of AICN (100 mM), DCI (100 mM) and methyl isonitrile (100 mM).

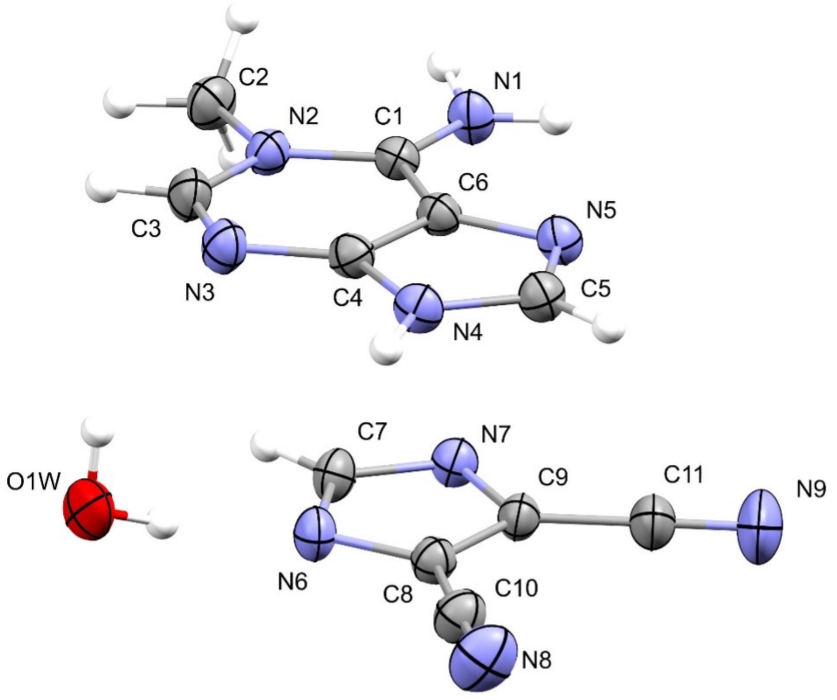

Figure S3. Displacement ellipsoid plot of 1-methyladenine (C1 to C6): 4,5-dicyanoimidazole (C7 to C11): H_2O crystal (50% probability).

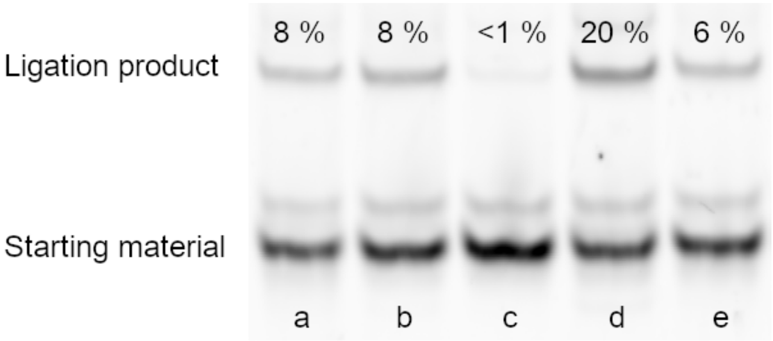

Figure S4. PAGE analysis of *in situ* nicked-duplex RNA ligation using 5′-pUACUGGCA-Cy3 (10 µM), 5′-GAGAACC (20 µM), 5′-CCAGUAGGUUCUC (10 µM), DCI (100 mM), $MgCl_2$ (10 mM), methyl isonitrile (400 mM).

a) with adenine (saturated);

b) with 1-methyladenine (saturated);

c) with AICA (saturated);

d) with 1-MeIm (100 mM);

e) with hypoxanthine (saturated).

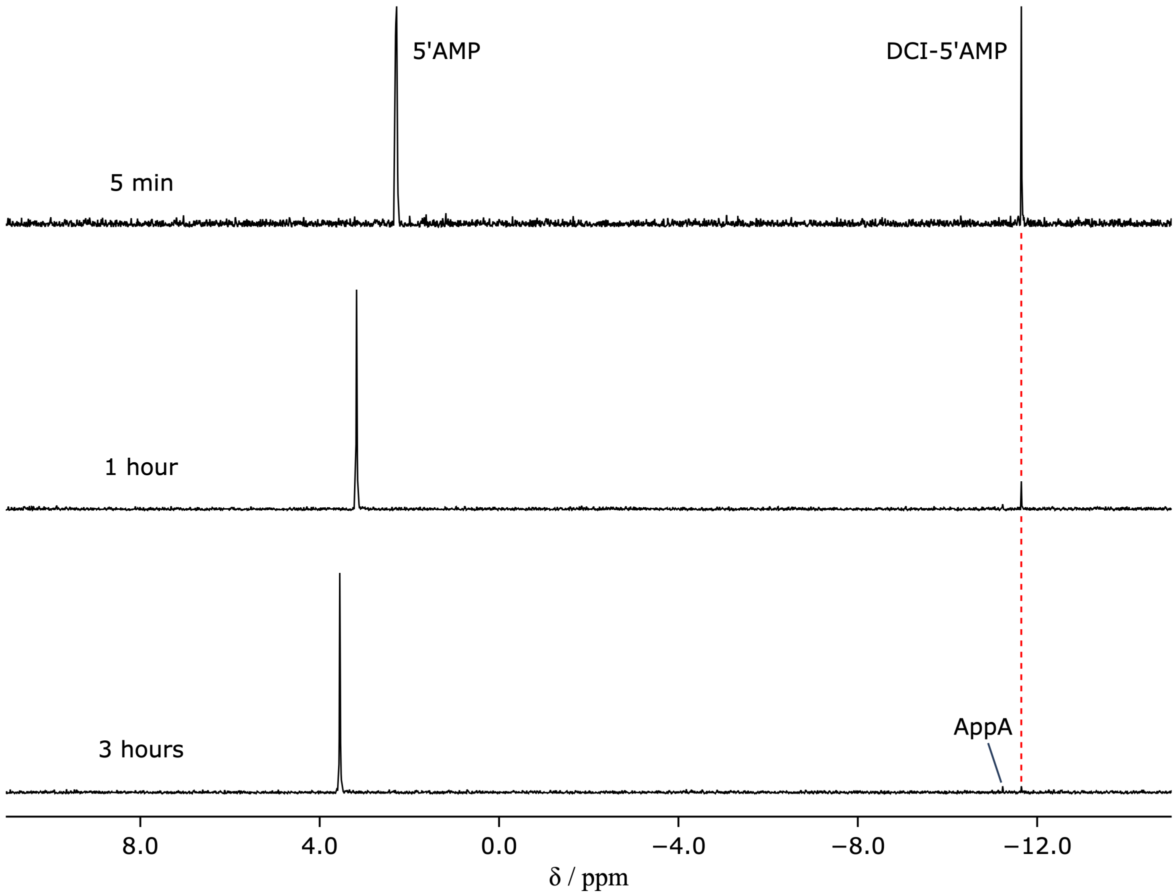

Figure S5. Stacked ^{31}P-NMR spectra of a solution of 5′-AMP (10 mM), $MgCl_2$ (10 mM), DCI (100 mM), acetaldehyde (100 mM), methyl isonitrile (100 mM).The chemical shift drift of the signal for 5′-AMP was caused by changing pH.

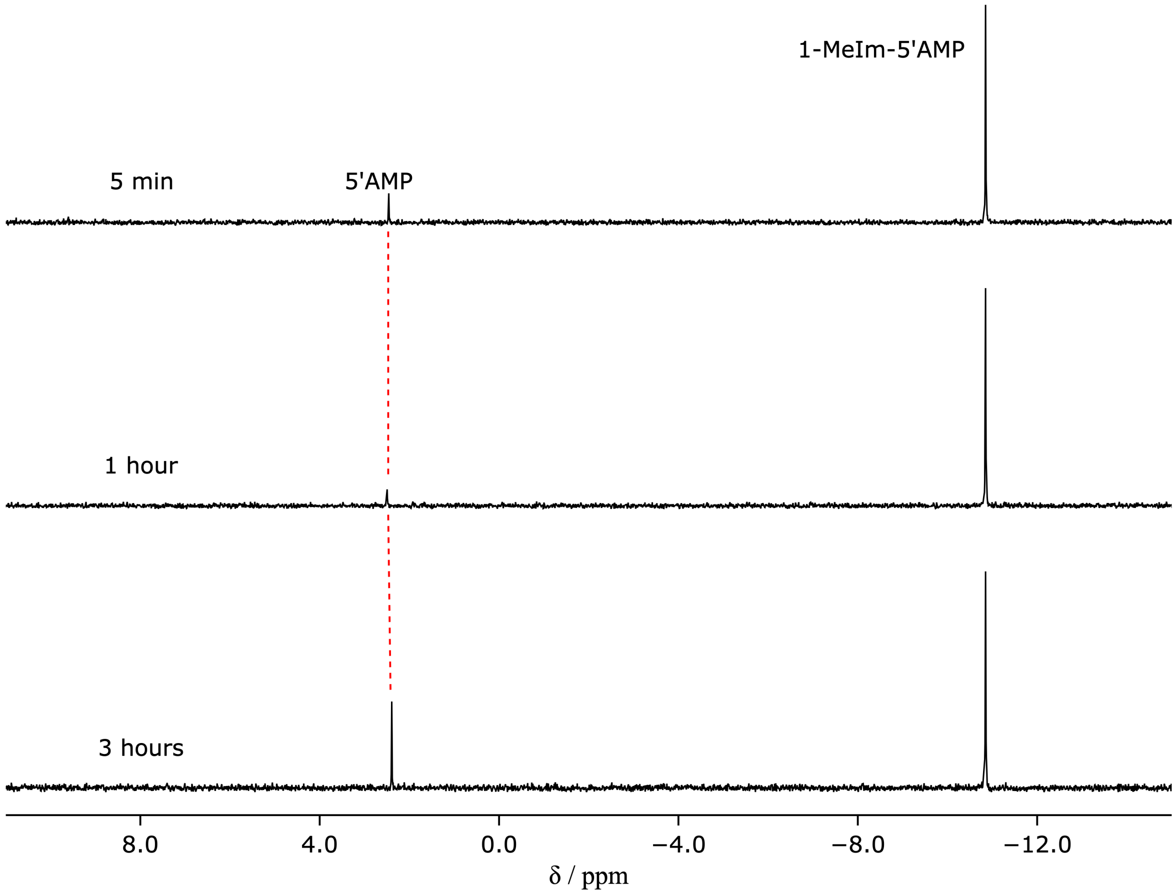

Figure S6. Stacked ^{31}P-NMR spectra of a solution of 5′-AMP (10 mM), $MgCl_2$ (10 mM), 1-MeIm (100 mM), acetaldehyde (100 mM), methyl isonitrile (100 mM).

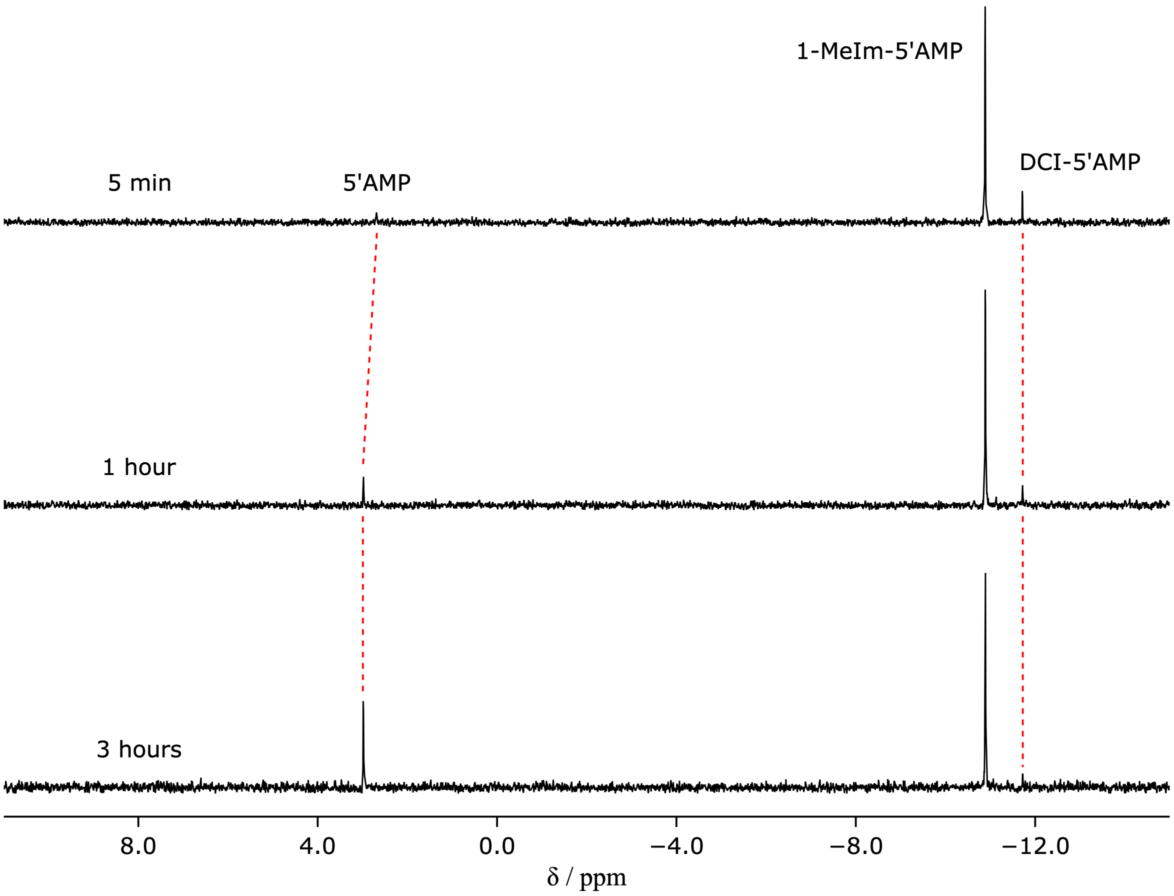

Figure S7. Stacked ^{31}P-NMR spectra of a solution of 5′-AMP (10 mM), $MgCl_2$ (10 mM), DCI (100 mM), 1-MeIm (100 mM), acetaldehyde (100 mM), methyl isonitrile (100 mM). The chemical shift drift of the signal for 5′-AMP was caused by changing pH.

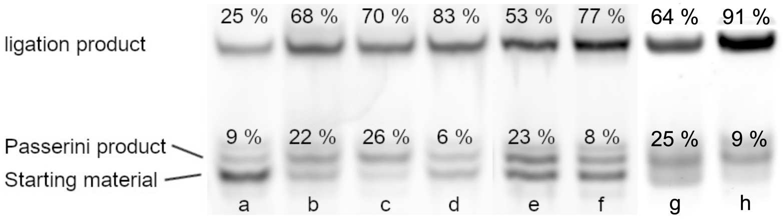

Figure S8. PAGE analysis of nicked-duplex RNA ligation using 5′-pUACUGGCA-Cy3 (10 µM), 5′-GAGAACC (20 µM), 5′-CCAGUAGGUUCUC (10 µM), acetaldehyde (400 mM), methyl isonitrile (400 mM), in different solutions at pH = 6, incubated for 24 hours.
a) with $MgCl_2$ (10 mM), 1-MeIm (100 mM), acetaldehyde (100 mM), methyl isonitrile (100 mM);
b) with $MgCl_2$ (10 mM), 1-MeIm (100 mM);
c) with $MgCl_2$ (10 mM), 1-MeIm (100 mM), DCI (10 mM);
d) with $MgCl_2$ (10 mM), 1-MeIm (100 mM), DCI (100 mM);
e) with $MnCl_2$ (10 mM), 1-MeIm (100 mM);
f) with $MnCl_2$ (10 mM), 1-MeIm (100 mM), DCI (100 mM);
g) with $MnCl_2$ (0.1 mM), 1-MeIm (100 mM), DCI (100 mM);
h) with $MnCl_2$ (1 mM), 1-MeIm (100 mM), DCI (100 mM).

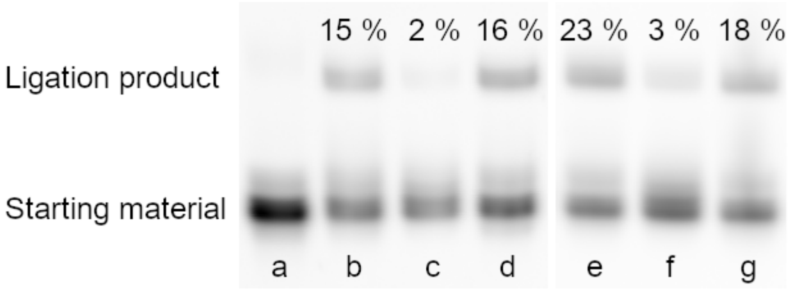

Figure S9. PAGE analysis of *in situ* nicked-duplex RNA ligation using 5′-pUACUGGCA-Cy3 (10 µM), 5′-GAGAACC (20 µM), 5′-CCAGUAGGUUCUC (10 µM), $MgCl_2$ (10 mM), KCN (50 mM), sodium hypochlorite (20 mM), pH value was 6.

a) before sodium hypochlorite was added;

b) with 1-MeIm (100 mM), incubated for 2 hours;

c) with DCI (50 mM), purine (50 mM), incubated for 2 hours;

d) with 1-MeIm (50 mM), DCI (50 mM), incubated for 2 hours;

e) same as b, incubated for 24 hours;

f) same as c, incubated for 24 hours;

g) same as d, incubated for 24 hours.

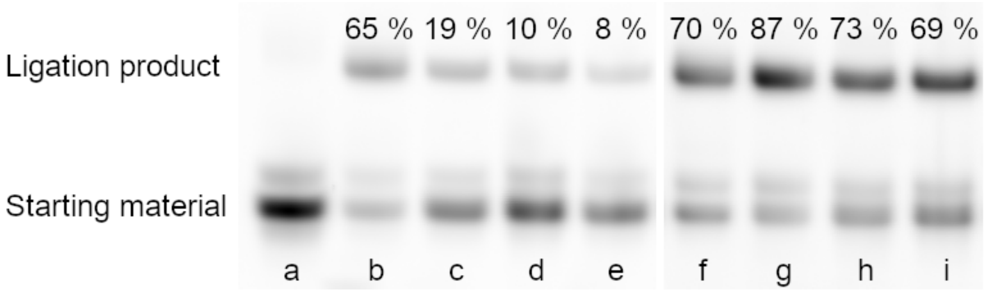

Figure S10. PAGE analysis of *in situ* nicked-duplex RNA ligation using 5′-pUACUGGCA-Cy3 (10 µM), 5′-GAGAACC (20 µM), 5′-CCAGUAGGUUCUC (10 µM), KCN (50 mM), $MnCl_2$ (10 mM), sodium hypochlorite (20 mM).
a) before sodium hypochlorite was added;
b) with 1-MeIm (100 mM), incubated for 2 hours;
c) with 1-MeIm (80 mM) and imidazole (20 mM), incubated for 2 hours;
d) with 1-MeIm (20 mM) and imidazole (80 mM), incubated for 2 hours;
e) with imidazole (100 mM), incubated for 2 hours;
f) same as b, incubated for 24 hours;
g) same as c, incubated for 24 hours;
h) same as d, incubated for 24 hours;
i) same as e, incubated for 24 hours.

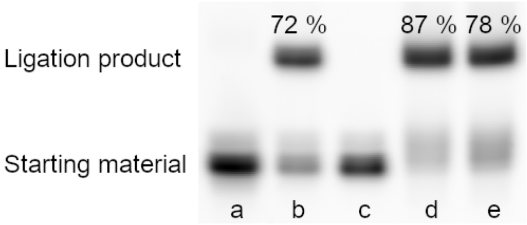

Figure S11. PAGE analysis of *in situ* nicked-duplex RNA ligation using 5′-pUACUGGCA-Cy3 (10 µM), 5′-GAGAACC (20 µM), 5′-CCAGUAGGUUCUC (10 µM), KCN (50 mM), $MnCl_2$ (10 mM).

a) before sodium hypochlorite was added;

b) with sodium hypochlorite (20 mM), incubated for 24 hours;

c) without sodium hypochlorite, incubated for 24 hours;

d) *N*-cyanoimidazole (100 mM) was added to the remaining mixture of b, then incubated for 24 hours;

e) *N*-cyanoimidazole (100 mM) was added to the remaining mixture of c, then incubated for 24 hours.

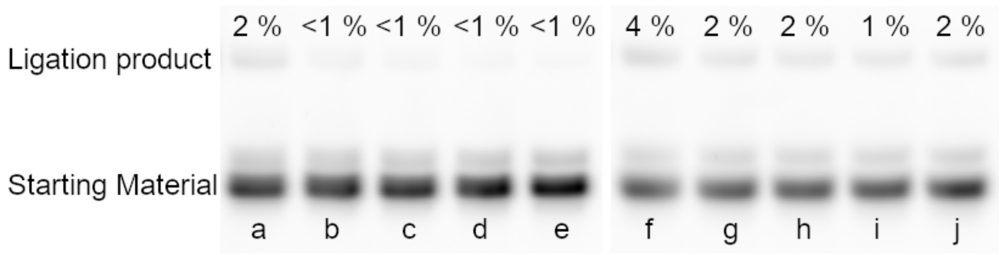

Figure S12. PAGE analysis of *in situ* nicked-duplex RNA ligation using 5′-pUACUGGCA-Cy3 (10 µM), 5′-GAGAACC (20 µM), 5′-CCAGUAGGUUCUC (10 µM), KCN (50 mM), $MgCl_2$ (10 mM), sodium hypochlorite (20 mM).

a) with 1-MeIm (80 mM) and imidazole (20 mM), incubated for 2 hours;

b) with 1-MeIm (60 mM) and imidazole (40 mM), incubated for 2 hours;

c) with 1-MeIm (40 mM) and imidazole (60 mM), incubated for 2 hours;

d) with 1-MeIm (20 mM) and imidazole (80 mM), incubated for 2 hours;

e) with imidazole (100 mM), incubated for 2 hours;

f) same as a, incubated for 24 hours;

g) same as b, incubated for 24 hours;

h) same as c, incubated for 24 hours;

i) same as d, incubated for 24 hours;

j) same as e, incubated for 24 hours.

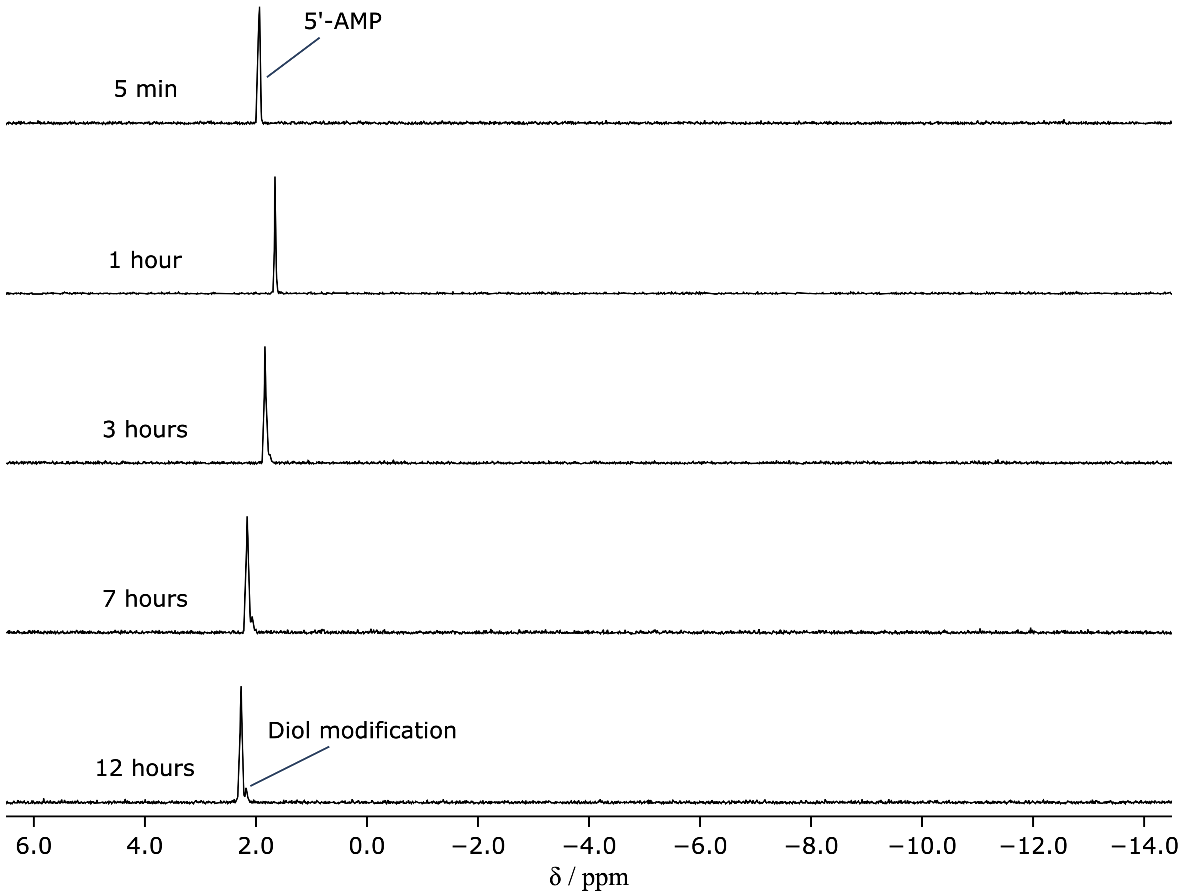

Figure S13. Stacked ^{31}P-NMR spectra of a solution of 5′-AMP (10 mM), imidazole (100 mM), potassium cyanide (50 mM), sodium hypochlorite (20 mM). The chemical shift drift of the signal for 5′-AMP was caused by changing pH.

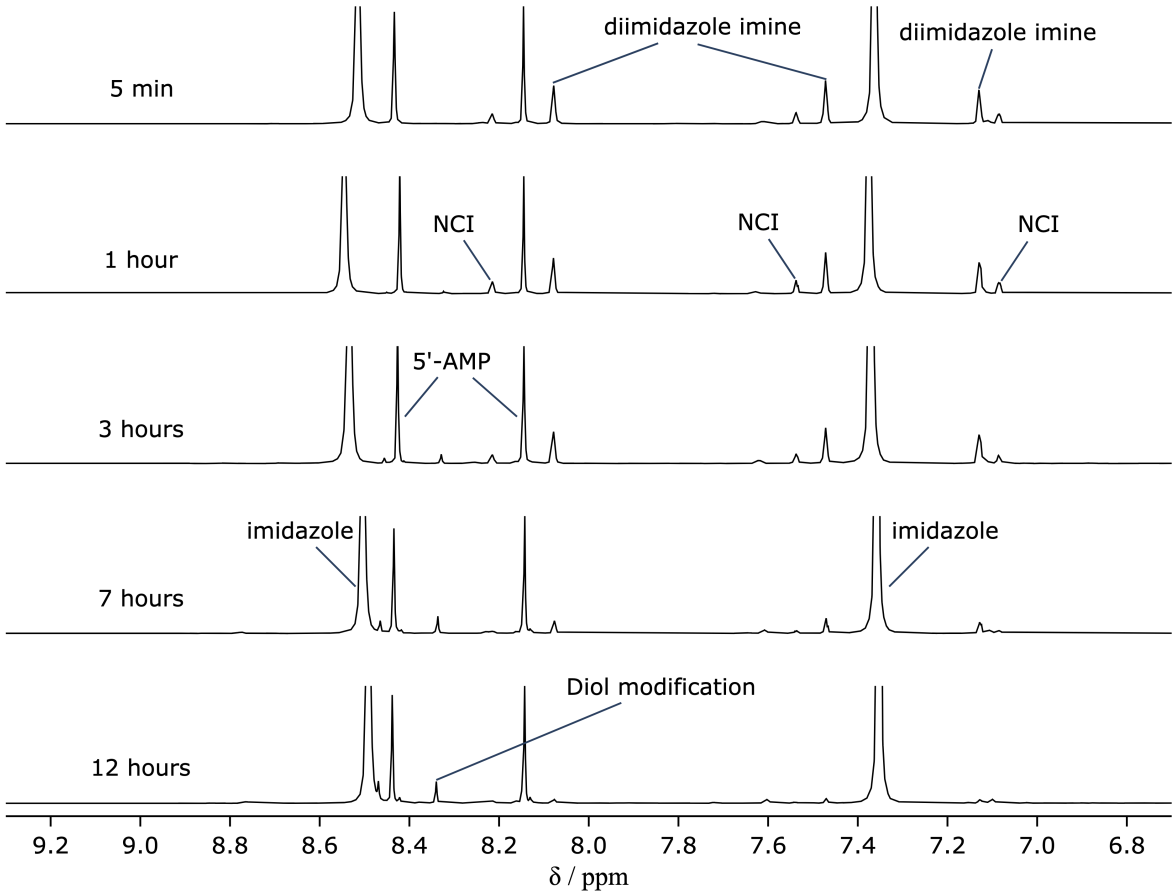

Figure S14. Stacked ^{1}H-NMR spectra of a solution of 5′-AMP (10 mM), imidazole (100 mM), potassium cyanide (50 mM), sodium hypochlorite (20 mM). The chemical shift drift of the signal for 5′-AMP was caused by changing pH.

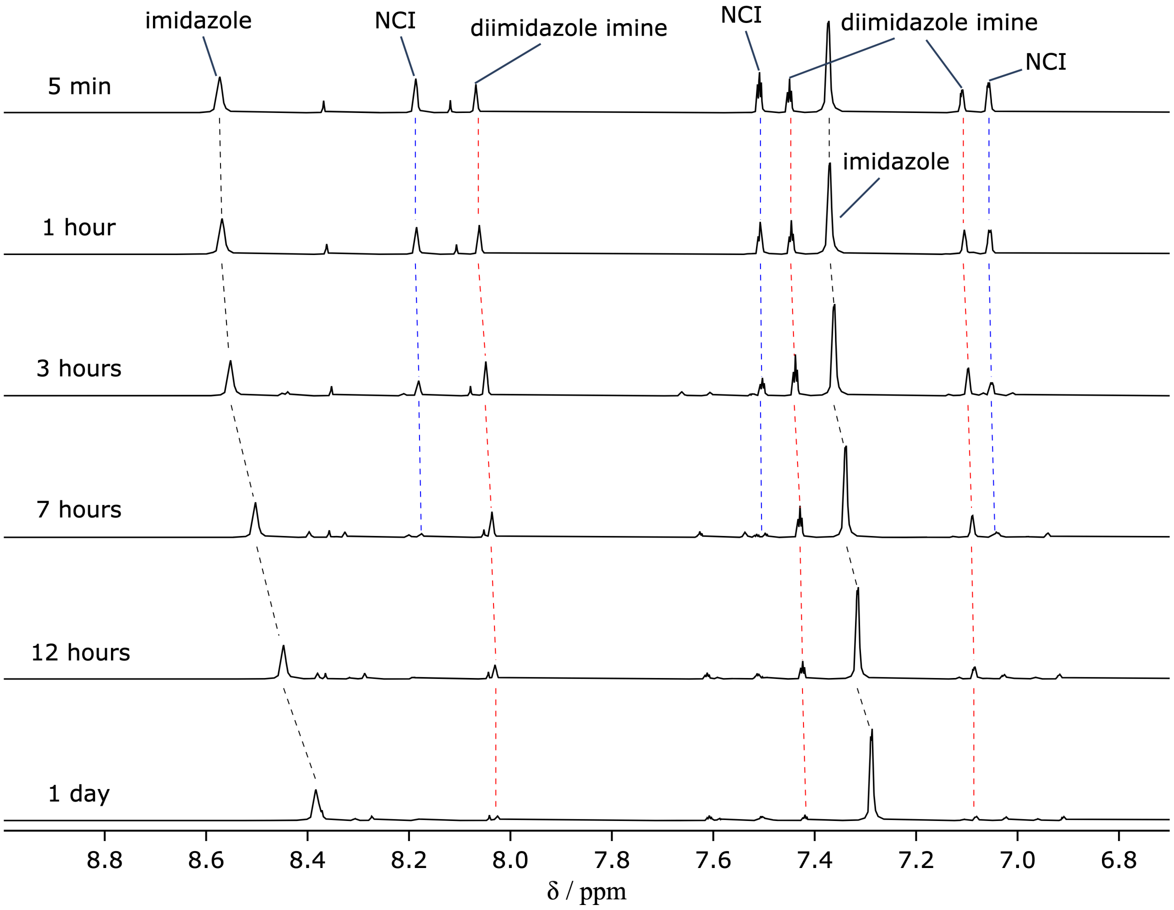

Figure S15. Stacked ^{1}H-NMR spectra of a solution of 5′-AMP (10 mM), imidazolium nitrate (200 mM, pH = 6.2), NCI (100 mM). The chemical shift drift of the signal for imidazole was caused by changing pH, after 1 day incubation, pH value was 6.6.

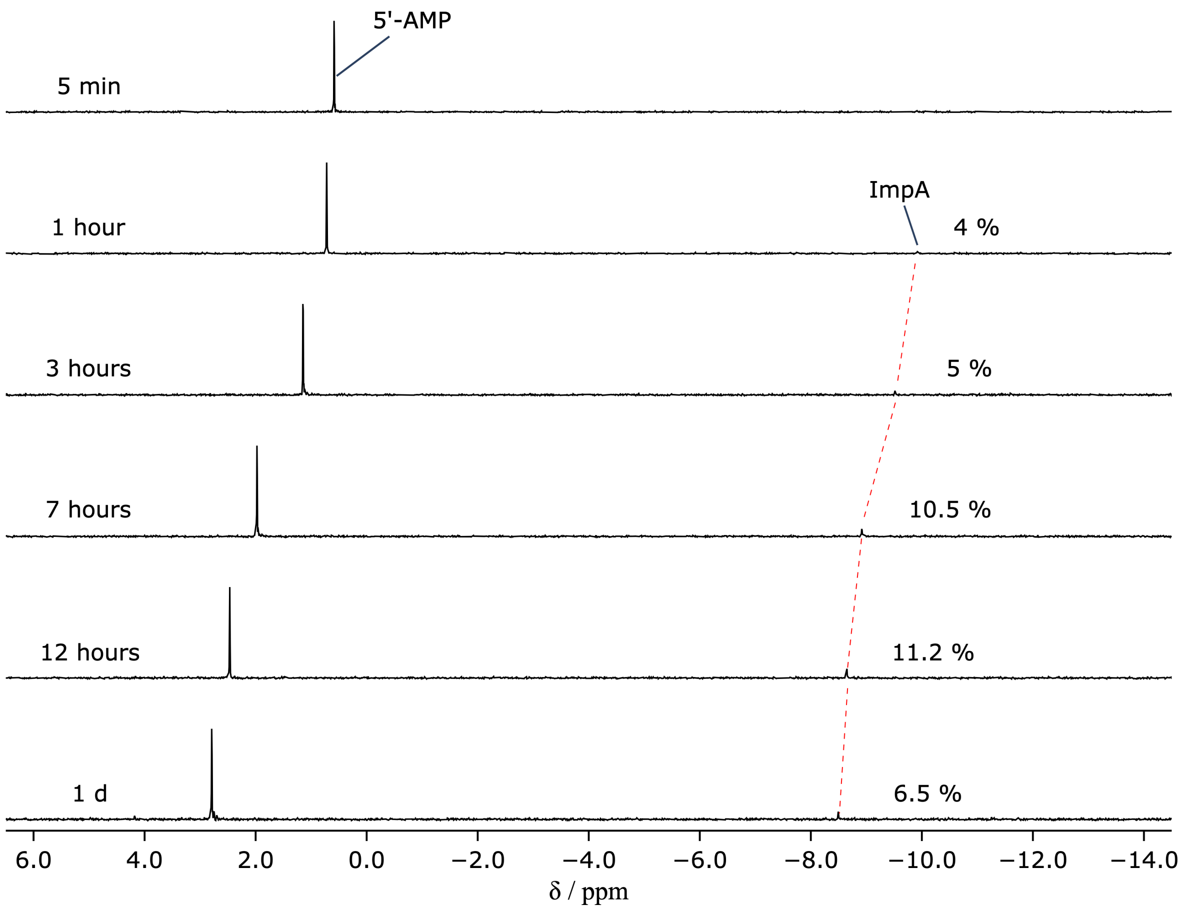

Figure S16. Stacked ^{31}P-NMR spectra of a solution of 5′-AMP (10 mM), imidazolium nitrate (200 mM, pH = 6.2), NCl (100 mM). The chemical shift drift of the signal for imidazole was caused by changing pH, after 1 day incubation, pH value was 6.6.

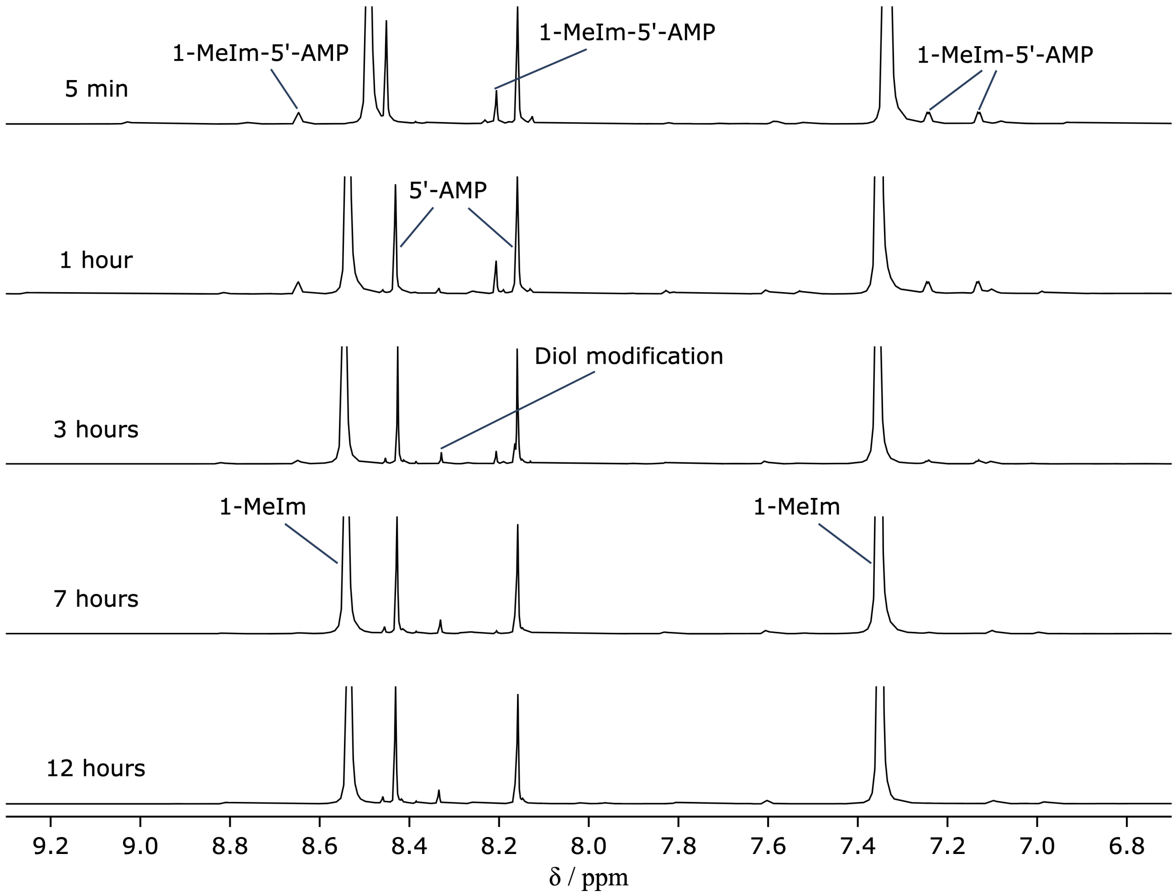

Figure S17. Stacked ^{1}H-NMR spectra of a solution of 5′-AMP (10 mM), 1-MeIm (100 mM), potassium cyanide (50 mM), sodium hypochlorite (20 mM). The chemical shift drift of the signal for 1-MeIm was caused by changing pH.

Table S1. Summary of crystal and refinement data

CCDC number	2302125
Cambridge data number	AB_B1_0019
Chemical formula	$C_{11}H_{11}N_9O$
Formula weight	285.29
Temperature / K	180(2)
Crystal system	monoclinic
Space group	$P\ 2_1/c$
a / Å	6.4786(2)
b / Å	15.6097(5)
c / Å	13.6164(5)
alpha / °	90
beta / °	100.478(2)
gamma / °	90
Unit-cell volume / $Å^3$	1354.05(8)
Z	4
Calc. density / g cm^{-3}	1.399
F(000)	592
Radiation type	Cu Kα
Absorption coefficient / mm^{-1}	0.844
Crystal size / mm^3	0.18 ×0.06 ×0.04
2-Theta range / °	8.70 – 133.86
Completeness to max 2-theta	0.999
No. of reflections measured	24685
No. of independent reflections	2407
R(int)	0.0402
No. parameters / restraints	211 / 0
Final R1 values ($I > 2\sigma(I)$)	0.0321
Final wR(F^2) values (all data)	0.0808
Goodness-of-fit on F^2	1.075
Largest difference peak hole / e $Å^{-3}$	0.144, -0.214

Table S2. Unprocessed gels for Figures

	Figure 1<br>Figure S10<br>Figure S12
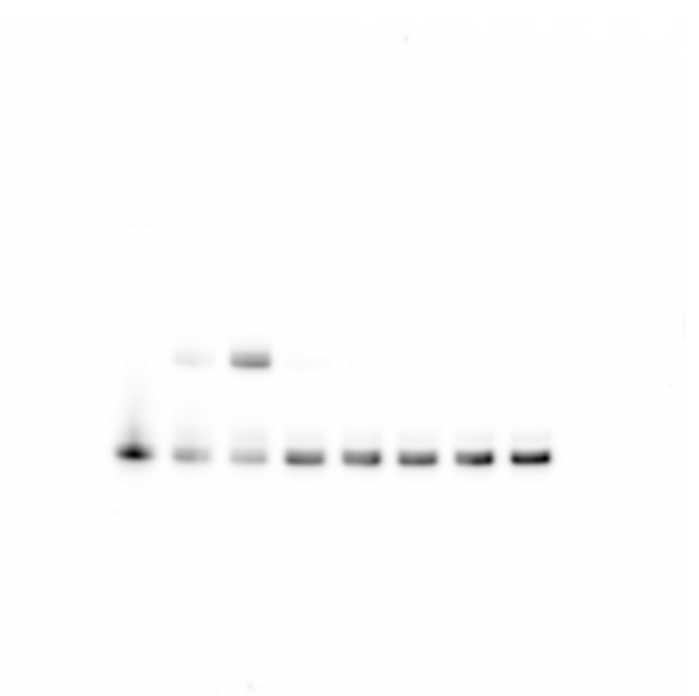	Figure 1<br>Figure S12

Continued on next page

Continued

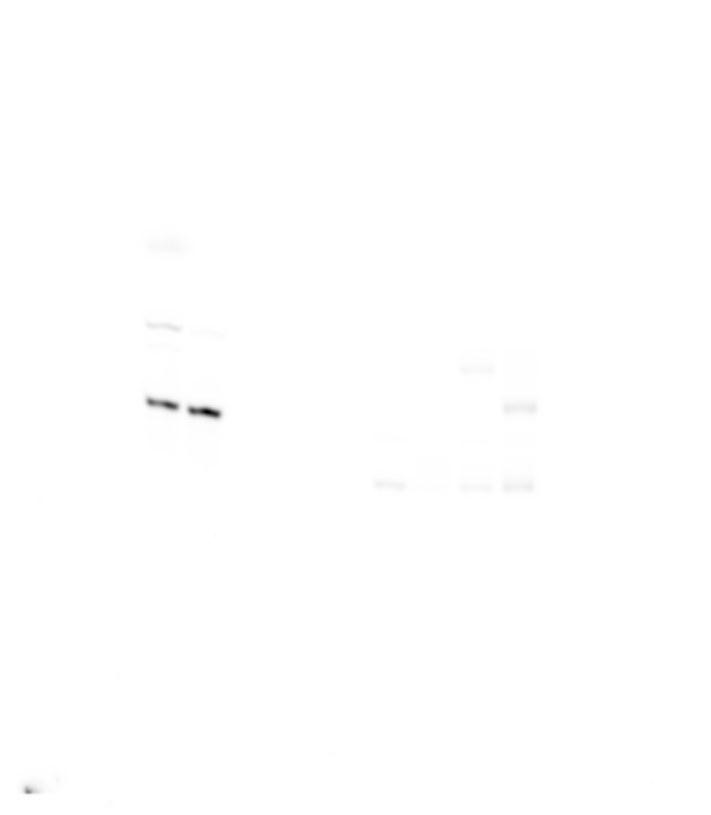

Figure S1

Continued on next page

Continued

Figure S4

Continued on next page

Continued

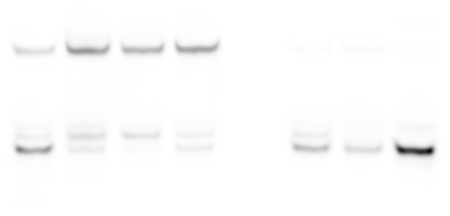

Figure S8

Continued on next page

Continued

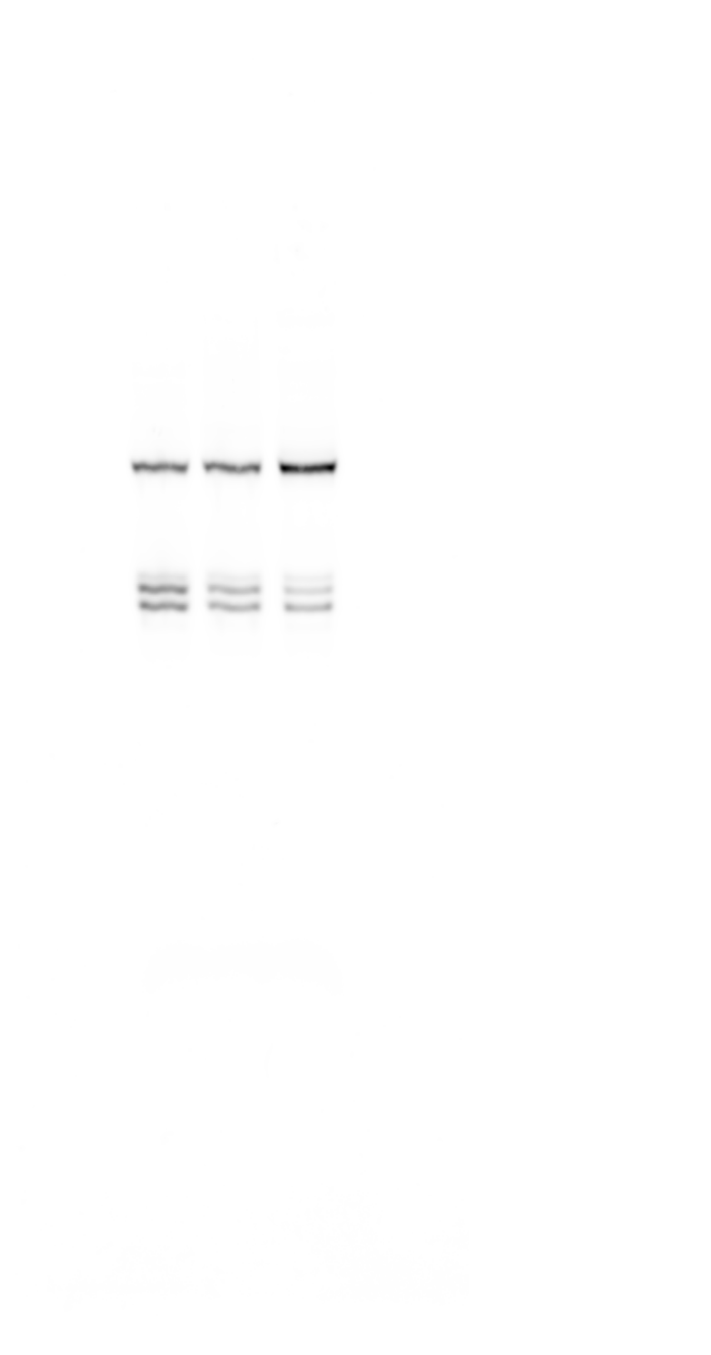

Figure S8

Continued on next page

Continued

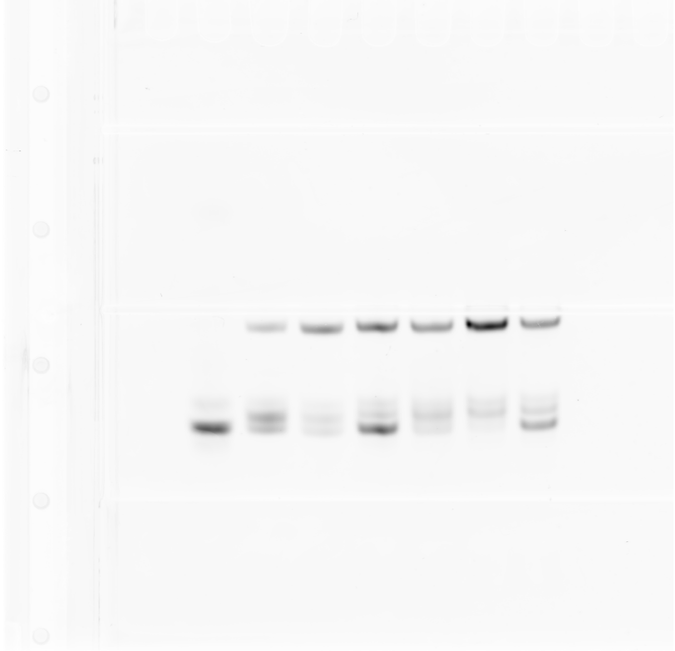

Figure S8

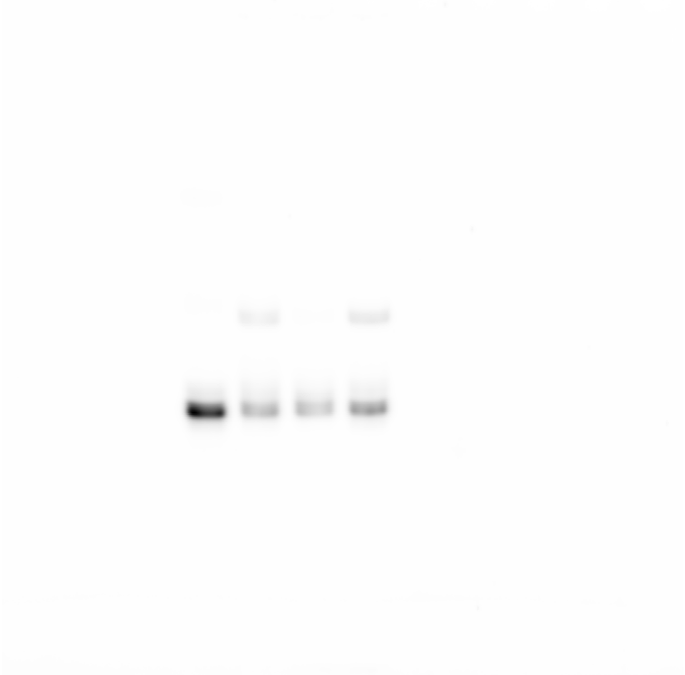

Figure S9

Continued on next page

Continued

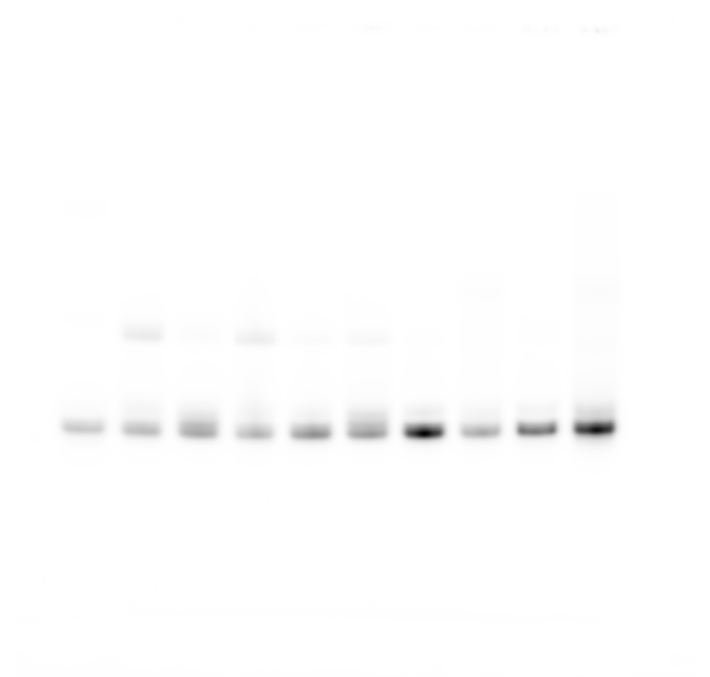

Figure S9

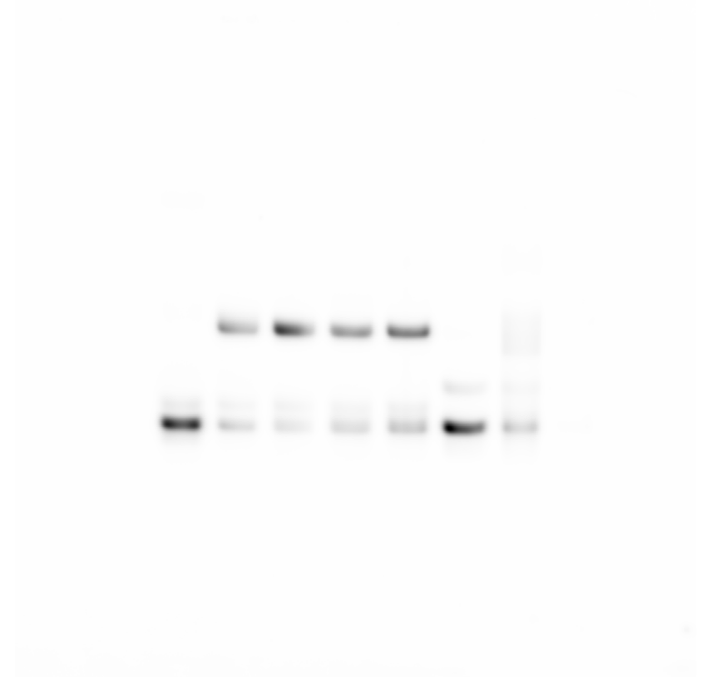

Figure S10

Continued on next page

Continued

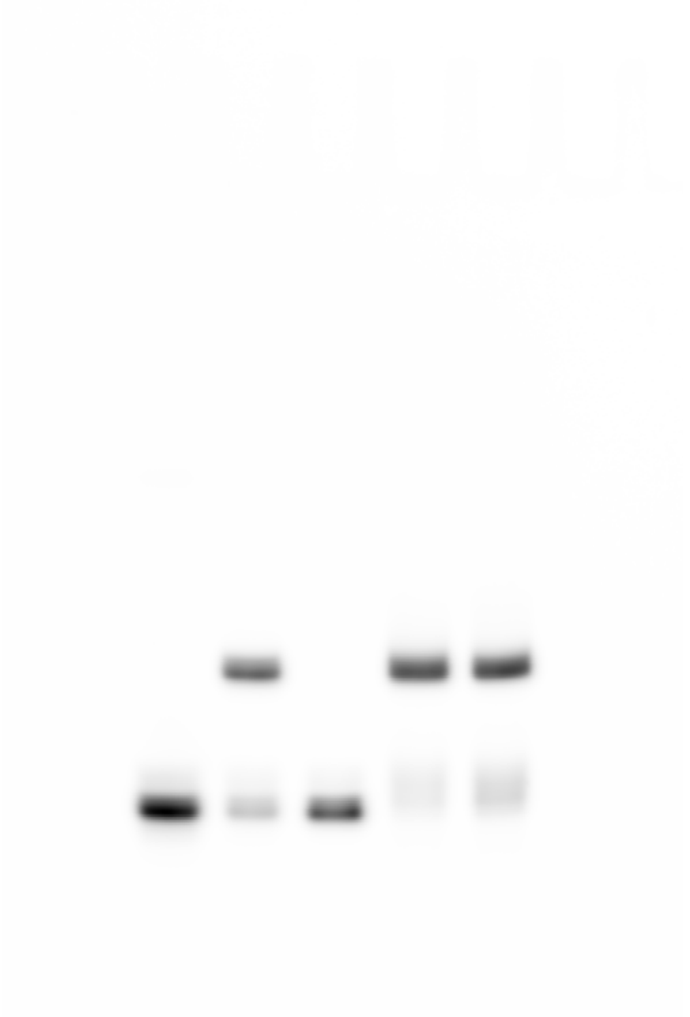

Figure S11

References

[1] Z. Liu, L.-F. Wu, J. Xu, C. Bonfio, D. A. Russell and J. D. Sutherland, *Nature chemistry*, 2020, **12**, 1023–1028.

[2] J. Xu, V. Chmela, N. J. Green, D. A. Russell, M. J. Janicki, R. W. Góra, R. Szabla, A. D. Bond and J. D. Sutherland, *Nature*, 2020, **582**, 60–66.

[3] J. Xu, N. J. Green, D. A. Russell, Z. Liu and J. D. Sutherland, *Journal of the American Chemical Society*, 2021, **143**, 14482–14486.

[4] L.-F. Wu, Z. Liu, S. J. Roberts, M. Su, J. W. Szostak and J. D. Sutherland, *Journal of the American Chemical Society*, 2022, **144**, 13920–13927.

[5] G. M. Sheldrick, *Acta Crystallographica Section A: Foundations and Advances*, 2015, **71**, 3–8.
